# Haplotype divergence supports long-term asexuality in the oribatid mite *Oppiella nova*

**DOI:** 10.1073/pnas.2101485118

**Published:** 2021-09-17

**Authors:** Alexander Brandt, Patrick Tran Van, Christian Bluhm, Yoann Anselmetti, Zoé Dumas, Emeric Figuet, Clémentine M. François, Nicolas Galtier, Bastian Heimburger, Kamil S. Jaron, Marjorie Labédan, Mark Maraun, Darren J. Parker, Marc Robinson-Rechavi, Ina Schaefer, Paul Simion, Stefan Scheu, Tanja Schwander, Jens Bast

**Affiliations:** ^a^Johann-Friedrich-Blumenbach Institute of Zoology and Anthropology, University of Goettingen, 37073 Goettingen, Germany;; ^b^Department of Ecology and Evolution, University of Lausanne, 1015 Lausanne, Switzerland;; ^c^Abteilung Boden und Umwelt, Forstliche Versuchs- und Forschungsanstalt Baden-Wuerttemberg, 79100 Freiburg, Germany;; ^d^Group Phylogeny and Molecular Evolution, Institut des Sciences de l’Evolution de Montpellier, 34090 Montpellier, France;; ^e^CoBIUS Lab, Department of Computer Science, University of Sherbrooke, Sherbrooke, QC J1K2R1, Canada;; ^f^Laboratoire d’Ecologie des Hydrosystèmes Naturels et Anthropisés, École Nationale des Travaux Publics de l’État, Université Claude Bernard Lyon 1, 69622 Villeurbanne, France;; ^g^Group Evolutionary Bioinformatics, Swiss Institute of Bioinformatics, 1015 Lausanne, Switzerland;; ^h^Institute of Evolutionary Biology, School of Biological Sciences, University of Edinburgh, Edinburgh EH9 3FL, United Kingdom;; ^i^Laboratory of Evolutionary Genetics and Ecology, Unit in Environmental and Evolutionary Biology, Université de Namur, 5000 Namur, Belgium;; ^j^Section Biodiversity and Ecology, Centre of Biodiversity and Sustainable Land Use, 37073 Goettingen, Germany;; ^k^Institute for Zoology, University of Cologne, 50674 Cologne, Germany

**Keywords:** Meselson effect, asexuality, haplotype divergence, oribatid mites

## Abstract

Sex strongly impacts genome evolution via recombination and segregation. In the absence of these processes, haplotypes within lineages of diploid organisms are predicted to accumulate mutations independently of each other and diverge over time. This so-called “Meselson effect” is regarded as a strong indicator of the long-term evolution under obligate asexuality. Here, we present genomic and transcriptomic data of three populations of the asexual oribatid mite species *Oppiella nova* and its sexual relative *Oppiella subpectinata*. We document strikingly different patterns of haplotype divergence between the two species, strongly supporting Meselson effect–like evolution and long-term asexuality in *O. nova*: I) variation within individuals exceeds variation between populations in *O. nova* but vice versa in *O. subpectinata*; II) two *O. nova* sublineages feature a high proportion of lineage-specific heterozygous single-nucleotide polymorphisms (SNPs), indicating that haplotypes continued to diverge after lineage separation; III) the deepest split in gene trees generally separates the two haplotypes in *O. nova*, but populations in *O. subpectinata*; and IV) the topologies of the two haplotype trees match each other. Our findings provide positive evidence for the absence of canonical sex over evolutionary time in *O. nova* and suggest that asexual oribatid mites can escape the dead-end fate usually associated with asexual lineages.

## Significance

Putatively ancient asexual species pose a challenge to theory because they appear to escape the predicted negative long-term consequences of asexuality. Although long-term asexuality is difficult to demonstrate, specific signatures of haplotype divergence, called the “Meselson effect,” are regarded as strong support for long-term asexuality. Here, we provide evidence for the Meselson effect in an asexual oribatid mite species, *Oppiella nova*, and we show that the effect is not caused by hybridization or polyploidization. Our findings provide conclusive evidence for the long-term absence of sex in *O. nova* and suggest that asexual oribatid mites can escape the dead-end fate usually associated with asexual reproduction.

Sexual reproduction is considered as a prerequisite for the long-term persistence of eukaryote species, because it reduces selective interference among loci and thus facilitates adaptation and purifying selection [recently reviewed in Otto (1)]. Contrary to this scientific consensus, some exceptional taxa appear to have persisted in the absence of sex over millions of years, the so-called “ancient asexual scandals” [sensu Judson and Normark (2)]. These exceptional taxa are invaluable, because by understanding how they persisted as asexuals they could help to identify the adaptive value of sex (3), one of the major riddles in evolutionary biology (4, 5). However, several species originally believed to be long-term asexuals were later suggested to be either recently derived asexuals or to engage in some form of rare or noncanonical sex (6–10). At least two candidates for long-term asexuality remain, the darwinulid ostracods and several parthenogenetic lineages of oribatid mites. Both groups appear to have persisted for tens of millions of years (11, 12) and diversified into ecologically different species (7, 13). However, support for obligate asexuality in darwinulid ostracods and oribatid mites is largely based on negative evidence, i.e., the absence or extreme rarity of males among thousands of females and the nonfunctionality of these rare males (14–17). Screening these groups for positive evidence of long-term asexuality is therefore of major importance.

**Fig. 1.**
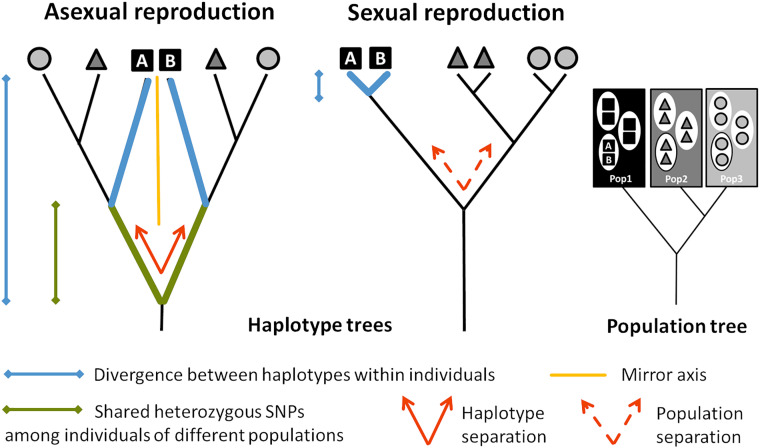
Nuclear haplotype trees expected under (long-term) obligate asexual and sexual reproduction. In diploid, functionally clonal organisms, homologous chromosomes accumulate mutations independently of each other and evolve as independent lineages (note that this can be restricted to specific regions sheltered from a loss of heterozygosity caused by mechanisms such as gene conversion). Accordingly, divergence between haplotypes within individuals (blue) is expected to exceed the mean divergence between haplotypes of individuals from different populations. Furthermore, the haplotype tree fully separates homologous haplotypes at its deepest split (red), which results in high frequency of heterozygous SNPs shared among individuals of different populations (green). Finally, the topologies of haplotype subtrees A and B are expected to match each other (the orange line represents the mirror axis) due to their parallel divergence. In sexual organisms, haplotype divergence is expected to follow population divergence and the haplotype tree to resemble that of the populations. Therefore, in sexuals, divergence between haplotypes within individuals is expected to be smaller than the divergence between populations, and the haplotype tree fully separates populations (red dashed). Adapted from Schwander et al. (18).

One of the strongest predictions for evolution without recombination and segregation is that the two haplotypes (each stemming from one homologous chromosome copy) within a diploid clonal lineage accumulate mutations independently of each other. Thus, after the loss of sex, the haplotype sequences diverge over time, and levels of intraindividual heterozygosity increase (Fig. 1). This intraindividual haplotype divergence is commonly known as the “Meselson effect” (19, 20).

Surprisingly, there has only been equivocal empirical validation of this strong theoretical prediction thus far. In several asexual lineages, the Meselson effect was not found [e.g., darwinulid ostracods (21)], or could be explained by mechanisms other than haplotype divergence after the transition to asexuality, such as a hybrid origin [e.g., *Meloidogyne* nematodes (6)] or divergence between paralogs rather than between alleles [e.g., bdelloid rotifers (22, 23) and *Timema* stick insects (18, 24); reviewed in Hoerandl et al. (25)]. Potential support for the Meselson effect was found in fissiparous species of *Dugesia* flatworms, but with data on only two genes, alternative explanations such as a hybrid origin or divergent paralogs could not be excluded (26). The as-yet strongest support comes from a whole-genome study of obligately asexual trypanosomes, unicellular parasitic flagellates, in which some genomic regions are highly heterozygous and show the expected parallel haplotype divergence (27). We still lack any support from genome-wide analyses of the Meselson effect in asexual animals.

One of the most promising eukaryotic systems for understanding long-term persistence in the absence of sex are oribatid mites (9, 11). Oribatid mites are small (150–1,400 μm), abundant and mostly soil-living chelicerates that play an important role as decomposers in most terrestrial ecosystems (11, 28, 29). A number of lineages lost sex independently, providing the possibility for comparative analyses (30–32). As yet, the cellular mechanism underlying asexuality in oribatid mites has not been determined with confidence. Cytological studies, focused mostly on a single species (*Archegozetes longisetosus*), have suggested a modified meiosis (holocentric chromosomes undergoing terminal fusion automixis with an inverted sequence of meiotic divisions) that preserves heterozygosity in regions sheltered from recombination and other homogenizing mechanisms (33–38).

In this study, we characterize haplotype divergence patterns in the asexual oribatid mite species *Oppiella nova* and its sexual relative *Oppiella subpectinata.* A previous study, based on molecular divergence estimates, suggested that *O. nova* persisted in the absence of sex for millions of years, given that sublineages within this species split 6–16 My ago (39, 40). Using de novo genomes and polymorphism data from transcriptome resequencing, we tested for four population genomic signatures expected under obligate asexuality (Meselson effect; Fig. 1). These signatures should be present in the asexual species *O. nova*, but absent in its close sexual relative *O. subpectinata*: I) high divergence within individuals, exceeding the divergence between populations (Fig. 1, blue); II) high frequency of shared heterozygous variants among individuals of different populations (indicating that haplotypes diverged prior to separation of populations; Fig. 1, green); III) the deepest split in allele phylogenies separates haplotypes, not populations (as opposed to sexual organisms where the deepest split typically separates populations; Fig. 1, red); IV) the topologies of trees based on haplotypes A and B match each other due to parallel divergence of haplotypes during population separation (Fig. 1, orange).

## Results

### De Novo Genomes.

We first de novo assembled genomes of single individuals of the asexual oribatid mite species *O. nova* and one of its closest known sexual relatives *O. subpectinata*. The quality- and contaminant-filtered assemblies (v03; *Data Availability*) spanned a total size of 197 Mb for *O. nova* and 213 Mb for *O. subpectinata*. They contained 63,118 and 60,250 scaffolds, with an N_50_ of 6,753 and 7,017 bp, with 23,761 and 23,555 genes annotated (see *SI Appendix*, Tables S1–S3 and *Methods* for details). Divergence estimates between single-copy orthologs of *O. nova* and *O. subpectinata* were large (p-distance: median 16.7%; based on 2,754 orthologs). Despite the low assembly contiguity (likely caused by whole-genome amplification prior to sequencing), the genomes were of sufficient quality for downstream analyses, which focused on patterns of heterozygosity and polymorphism. This is reflected by the high completeness scores of arthropod core genes (C) as inferred via BUSCO, with few fragmented (F), missing (M), or duplicated (D) genes (C: 87.5%, F: 6.6%, M: 5.9%, D: 8.6% for *O. nova*; and C: 86.2%, F: 6.4%, M: 7.4%, D: 7.9% for *O. subpectinata*; *SI Appendix*, Table S1).

#### I. The divergence within asexual individuals is large and exceeds the divergence between populations.

**Fig. 2.**
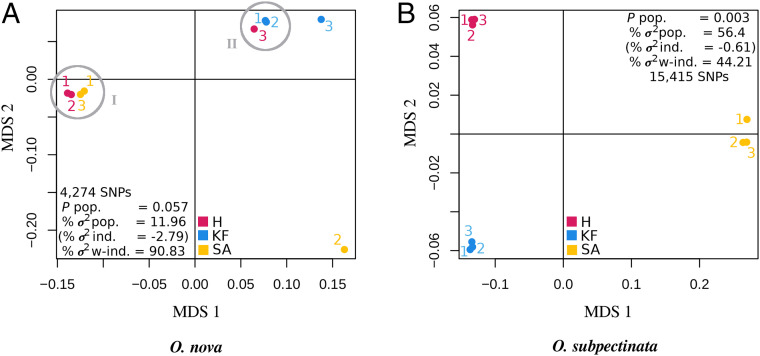
Genetic divergence is more extensive within individuals than between populations for the asexual *O. nova* (*A*), in contrast to the sexual *O. subpectinata* (*B*). In *O. nova*, there are multiple genetic lineages grouping individuals from different geographical locations. Lineages are represented by two clusters and two single individuals (lineages one and two highlighted by gray circles; nonsignificant between-population variation; rand-test *P* pop. = 0.057). Two *O. nova* individuals, individual 3 from location KF and individual 2 from location SA, are rather homozygous and likely do not feature the Meselson effect, while the remaining individuals do (*Results*, sections II–IV). Individuals of the sexual *O. subpectinata* clustered by location (significant between-population variation; rand-test *P* pop. = 0.003). Notably, the majority of total genetic variation is explained by differences between populations (% *σ*^2^ pop.) in *O. subpectinata*, but by within-individual differences in *O. nova* (% *σ*^2^ w-ind.; % *σ*^2^ ind.: % variation between individuals within location).

We analyzed within-individual and between-population divergence of individuals from three geographically distant locations in Germany (Hainich, H; Kranichstein Forest, KF; Schwäbische Alb, SA), using transcriptome data from three individuals per species and location (for sampling sites, see *SI Appendix*, Fig. S1 and *Methods*). Polymorphism data were generated by mapping transcriptome reads of each individual to the corresponding reference genome. For *O. nova*, a multidimensional scaling plot (MDS) (based on raw Hamming distances) revealed the presence of at least two clusters (hereafter referred to as divergent lineages), grouping individuals from different sampling locations (Fig. 2*A*). For *O*. *subpectinata*, individuals were separated into three distinct clusters, each corresponding to one location (Fig. 2*B*). Accordingly, between-location variation contributed much less to the species-wide genetic variation in *O. nova* (12%) than in *O. subpectinata* (56.4%). In *O. nova*, most variation (90.8%) was explained by variation within individuals.

Consistent with the large proportion of intraindividual variation in *O. nova*, heterozygosity for most (seven out of nine) *O. nova* individuals was higher than heterozygosity for eight of the nine *O. subpectinata* individuals (Table 1). This is the expected pattern given that haplotype divergence under functionally mitotic asexuality should result in increased heterozygosity levels compared to closely related sexual species, unless gene conversion or other homogenizing mechanisms occur at higher rates than new mutations (19). Notably, such homogenizing mechanisms are the most likely explanation for the heterozygosity differences among lineages in *O. nova* (*SI Appendix*, Fig. S2). Ongoing loss of heterozygosity constrains maximum haplotype divergence and could thus also explain the rather small heterozygosity difference between the seven heterozygous *O. nova* individuals and *O. subpectinata* (mean heterozygosity, 1.05% and 0.65%, respectively).

**Table 1.** Individual heterozygosity estimates as percentages of heterozygous sites among all sites with available SNP genotypes for all nine individuals

	*O. nova* (126,196 sites)		*O. subpectinata* (355,249 sites)	
Location	Individual	% heterozygous sites	Individual	% heterozygous sites
Hainich	H1	1.273	H1	0.619
	H2	1.285	H2	0.665
	H3	0.741	H3	0.640
Kranichstein forest	KF1	0.746	KF1	0.599
	KF2	0.771	KF2	0.581
	KF3	0.414	KF3	0.591
Schwäbische Alb	SA1	1.268	SA1	0.723
	SA2	0.441	SA2	0.685
	SA3	1.288	SA3	0.743

Inferred from transcriptome data; see also *SI Appendix*, Fig. S2.

#### II. An excess of lineage-specific heterozygous single-nucleotide polymorphisms indicates that haplotypes continued to diverge after lineage separation.

The relation between individual heterozygosity and (sub)population allele frequencies is expected to differ between obligate asexual organisms and panmictic sexual populations, with higher individual heterozygosity relative to population allele frequencies in asexuals (7, 41). We compared observed individual heterozygosity vs. heterozygosity expected from allele frequencies using F_is_ as a measure. We based estimates of expected heterozygosity on genetically differentiated locations in *O. subpectinata* but on genetically differentiated lineages (I + II) in *O. nova*, because genetic differentiation between locations was low in *O. nova* (Fig. 2*A*). F_is_ values were strongly negative for *O. nova* individuals (mean F_is_ = −0.328, Table 2). Such negative F_is_ values indicate an excess of observed heterozygosity, as expected for functionally clonal organisms. For *O. subpectinata*, values were in the range expected for a non-inbred panmictic sexual species (mean F_is_ = 0.002; Table 2) and values for *O. nova* were significantly lower (mean F_is_ = −0.328; Wilcox rank sum test, *W* = 0; *P* < 0.001).

**Table 2.** Per individual inbreeding coefficient estimates (F_is_)

	*O. nova*		*O. subpectinata*	
Location	Individual	F_is_ (no. sites)	Individual	F_is_ (no. sites)
Hainich	H1	−0.390 (5,414)	H1	0.015 (12,730)
	H2	−0.361 (5,414)	H2	−0.034 (12,730)
	H3	***−0.243 (3,471)***	H3	0.001 (12,730)
Kranichstein forest	KF1	***−0.246 (3,471)***	KF1	−0.006 (40,145)
	KF2	***−0.296 (3,471)***	KF2	0.023 (40,145)
	KF3	NA	KF3	0.002 (40,145)
Schwäbische Alb	SA1	−0.361 (5,414)	SA1	−0.018 (10,197)
	SA2	NA	SA2	0.058 (10,197)
	SA3	−0.398 (5,414)	SA3	−0.019 (10,197)

Estimates of F_is_ were based on location in *O. subpectinata*, but on genetically divergent lineages in *O. nova* (lineages I + II; lineage II, bold and italicized). Note that it was not possible to estimate F_is_ for *O. nova* KF3 and SA2 because they likely represent divergent lineages on their own, and estimating F_is_ requires a (sub)population context.

The extensive heterozygosity variation among *O. nova* individuals suggests that within a single origin of asexuality, the evolution of heterozygosity can follow strikingly different trajectories in different lineages. Independently of the potential causes driving the heterozygosity loss in some *O. nova* lineages (represented by individuals KF3, SA2), it is important to note that highly homozygous lineages cannot feature the Meselson effect as the rate of heterozygosity loss is greater or equivalent to the gain of heterozygosity via new mutations. We therefore conducted explicit tests for the Meselson effect solely on the seven of the nine individuals where it could potentially be present.

**Fig. 3.**
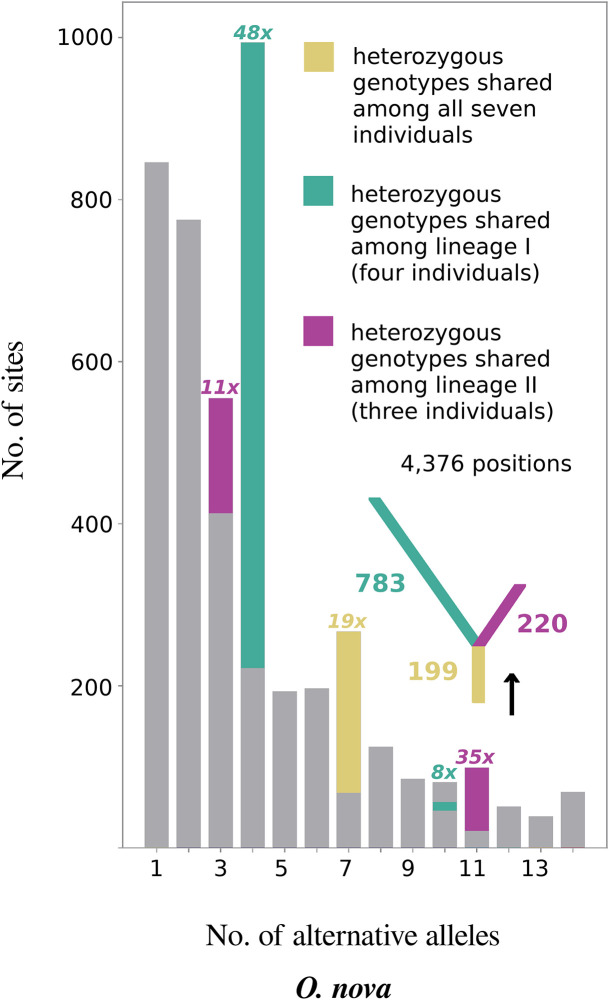
Excess of shared heterozygous SNPs among individuals of different populations and lineages for the asexual species *O. nova*. The site frequency spectrum (SFS) depicts the number of sites with a given number of nonreference variants over the seven heterozygous individuals (e.g., seven diploid individuals can display a maximum of 14 variants relative to the reference genome). Heterozygous genotypes shared among all seven individuals, or among individuals of lineages I and II privately, are color-highlighted and their excess over HWE indicated (8 to 48 times as frequent as expected under HWE; see legend). The SFS is consistent with the accumulation of shared heterozygous variants after the loss of sex, followed by lineage separation and independent accumulation of further heterozygosity in each lineage (*Inset* tree with numbers of shared heterozygous SNPs at each branch).

If the loss of sex in *O. nova* occurred prior to population separation, the observed heterozygosity excess in seven individuals is expected to result from shared heterozygous single-nucleotide polymorphisms (SNPs) among individuals of the two different lineages (green lines in Fig. 1). To test this, we generated a site frequency spectrum (SFS). Sites with heterozygous SNPs shared among the seven individuals were 19 times as frequent as expected under Hardy–Weinberg equilibrium (HWE) (yellow bar in Fig. 3). Furthermore, there was an excess of sites with heterozygous SNPs exclusively shared among the four individuals of lineage I (48 and 8 times as frequent as expected under HWE; turquoise bars in Fig. 3) or among the three individuals of lineage II (11 and 35 times as frequent as expected under HWE; purple bars in Fig. 3). These results are consistent with the accumulation of heterozygous variants after the loss of sex, followed by lineage divergence and independent accumulation of heterozygous variants within lineages I and II (*Inset* tree, Fig. 3).

#### III. The deepest split in many haplotype phylogenies separates haplotypes.

A classical signature of haplotype divergence under asexuality is the full separation of haplotypes A and B at the deepest split of a haplotype tree. By contrast, haplotypes are generally expected to diverge according to population divergence in sexual organisms (Fig. 1). To test these predictions, we phased haplotypes of the seven heterozygous individuals from the two genetic lineages of *O. nova* (which potentially show the Meselson effect based on percent heterozygous sites and F_is_ estimates; see above) and the nine individuals of *O. subpectinata* using the RNA-seq polymorphism data. We based all analyses on genomic regions with phases that formed a continuous overlap by at least 100 bp between at least two individuals (*Methods*). For *O. nova*, a total of 281 genome regions (median length, 358 bp) were phased, spanning 140,966 bp and representing 86.3% of 163,418 theoretically phaseable sites (genotypes with coverage ≥10 for all seven individuals; for detailed information on phaseable regions, see *SI Appendix*, Table S4 and *Methods*). For *O. subpectinata*, a total of 275 regions (median length, 563 bp) were phased. The regions spanned 206,255 bp, representing 58.1% of 355,249 theoretically phaseable sites, consistent with the considerably lower heterozygosity compared to *O. nova*.

Several previous studies suggested the Meselson effect on the basis of divergence between paralogs [as present, e.g., in polyploid lineages (22)] rather than alleles (22–25). To exclude that our findings are explained by paralog divergence, we confirmed that the phased regions represent allele haplotypes and not paralogs using coverage comparisons. We verified that the genomic read coverage of phaseable regions was not exceeding the coverage of single-copy genes from the BUSCO arthropod database (paralogs should have an at least 2× higher coverage; *Methods*). This was indeed the case as single-copy BUSCO genes, phased regions, and the scaffolds from which phased regions derived showed a median coverage of 126×, 101×, and 87×, respectively. By contrast, the median coverage of known duplicate BUSCO genes, which served as a positive control, was about twofold as high (207×; *SI Appendix*, Fig. S3).

**Fig. 4.**
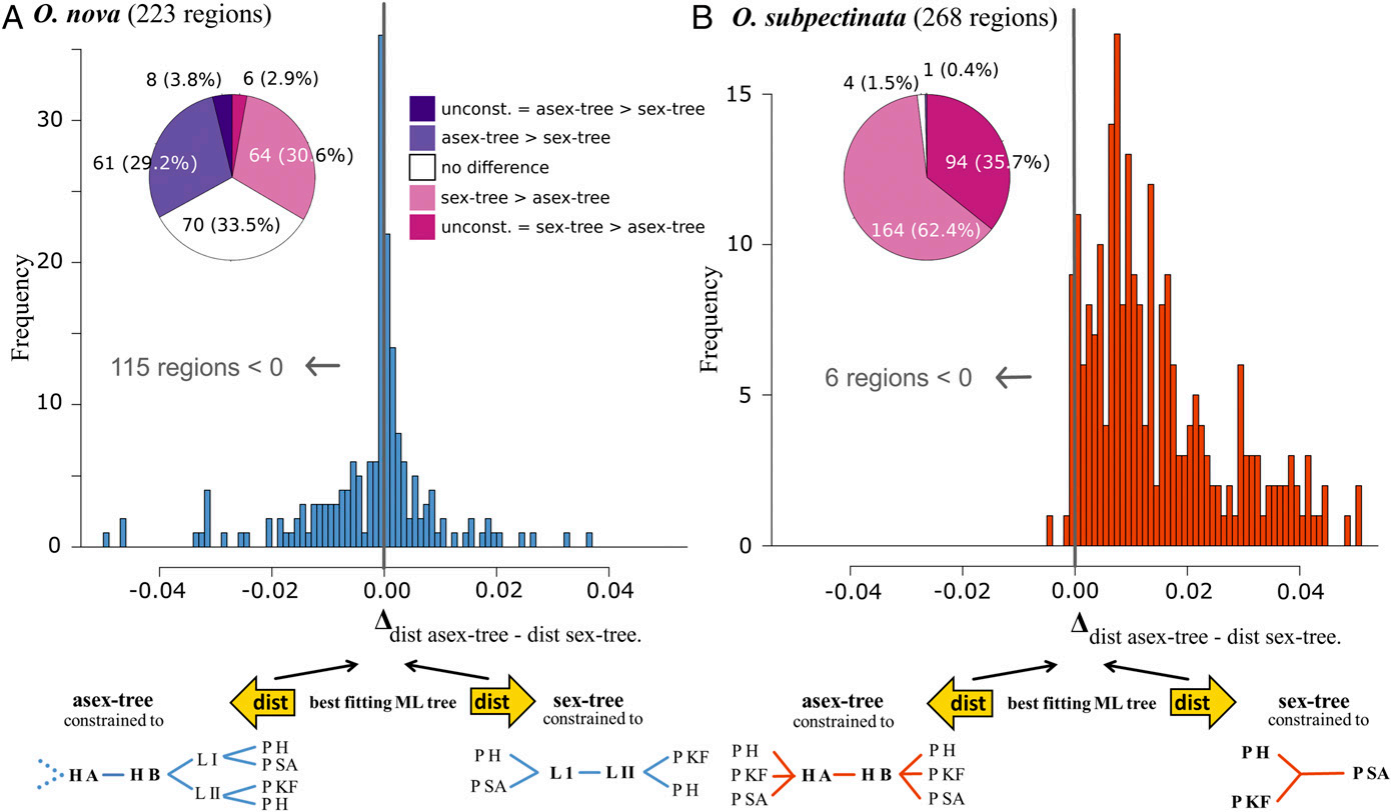
Haplotype trees are more consistent with asexuality in *O. nova* (*A*) but with sex in *O. subpectinata* (*B*). Frequency distribution of per-region tree-distance score comparisons (Δ _dist._ _asex-tree__−__dist._ _sex-tree_). The score measures the combined distance (dist) in topology and branch lengths between an unconstrained tree and one of two constrained trees (asex-tree, sex-tree; see schematic trees for each species, respectively). A negative value indicates that a phaseable region’s best ML tree is more similar to its asex-tree than to its sex-tree. Reconstruction of constrained trees was possible for regions with four or more unique aligned sequences present, i.e., 223 and 268 regions for *O. nova* and *O. subpectinata*, respectively (*Methods*). To improve legibility, the histogram ranges are limited from −0.05 to 0.05, thereby excluding 26 regions below and 8 regions above this range for *O. nova*, and 1 region below and 32 regions above the range for *O. subpectinata*. H A, haplotype A; H B, haplotype B; L I, lineage I; L II, lineage II; P H, population Hainich; P KF, population Kranichstein forest; P SA, population Schwäbische Alb; dashed branches, lineage separation followed by population separation as for haplotype B. *Inset* pie charts display topology categories (counts and percentages) based on tree topology tests, sorted from most to least Meselson effect-like topology, i.e., no difference in fit between the unconstrained tree and the asex-tree but rejection of the sex-tree (unconst. = asex-tree > sex-tree), rejection of the sex-tree when compared only to the asex-tree (asex-tree > sex-tree), no difference in fit between asex-tree and sex-tree (no difference), rejection of the asex-tree when compared only to the sex-tree (sex-tree > asex-tree), no difference in fit between the unconstrained tree and the sex-tree but rejection of the asex-tree (unconst. = sex-tree > asex-tree). For some phased regions, AU tests could not be run due to insufficient variation between haplotypes (14 regions in *O. nova*; 5 regions in *O. subpectinata*).

We next aligned the phased haplotype sequences and calculated best-fitting maximum likelihood (ML) trees. We then computed for each tree how distinct it was from two topology-constrained trees [based on comparing the lengths of branches separating all possible partitions between two trees, also referred to as “branch score distance” (42)]: 1) fully separating haplotypes A and B at the deepest split of the haplotype phylogeny, followed by population separation (consistent with predicted haplotype divergence under asexuality; hereafter referred to as “asex-tree”; Figs. 1 and 4*A*), and 2) separating haplotypes according to population divergence as expected for a sexual organism (hereafter referred to as “sex-tree”; Figs. 1 and 4*B*). We accounted for the observed coexistence of lineages I + II in *O. nova* by introducing lineage as an additional divergence level (Fig. 4*A*, blue trees; for *O. subpectinata* red trees). The Delta of the two tree distance scores is indicative of a phaseable region being more consistent with haplotype divergence under asexuality (Δ _dist._ _asex-tree__-__dist._ _sex-tree_ < 0) or sexuality (Δ _dist._ _asex-tree__-__dist._ _sex-tree_ > 0). For *O. nova*, 115 regions (51.6%) showed a Delta < 0, while for the sexual *O. subpectinata*, this applied for only six regions (2.2%; Fig. 4). These results are corroborated by tree topology tests showing 69 of 223 phaseable regions being significantly more consistent with the asex-tree in *O. nova* (including eight regions showing no difference in fit between the best fitting ML tree and the asex-tree; Fig. 4, *Insets*; *SI Appendix*, Table S5 and *Methods*). The 69 regions of *O. nova* spanned a total of 37,693 of the 163,418 theoretically phaseable sites, indicating that ∼23.1% of the *O. nova* transcriptome shows a significantly better fit with the expected haplotype divergence pattern under asexuality than under sexual reproduction.

#### IV. Matching of topologies of haplotype subtrees.

The high frequency of heterozygous genotypes shared among the seven individuals (Fig. 3) and the split of haplotypes A and B at the base of a haplotype tree (Fig. 4*A*) could theoretically be explained by hybridization at the origin of asexuality, while the excess of heterozygous SNPs private to lineages I or II could have been caused by the loss of heterozygosity at different sites within each lineage. Alternatively, lineages I and II could represent independent origins of asexuality via different hybridization events between closely related sexual species. We therefore tested for signatures of long-term asexuality following such putative hybridization events, by assessing parallel divergence of haplotypes within lineages I and II (comprising four individuals from populations H and SA and three individuals from populations H and KF). Specifically, while hybridization may bring ancestral versions of haplotypes A and B together in new asexual lineages, it does not explain subsequent parallel diversification of haplotypes A and B, which would have occurred after the ancestral hybridization event(s) (*SI Appendix*, Fig. S4). Thirty-one out of 39 resolved trees within lineage I fully separated haplotypes A and B, and 8 out of the 31 featured parallel divergence of haplotypes following the split. The 8 instances of parallel divergence are about four times as frequent as expected by chance, indicating that parallel divergence is a significant feature of lineage I (*P* < 0.001). For lineage II, 55 out of 90 resolved trees fully separated haplotypes, and 38 out of 55 featured parallel divergence. The 38 instances of parallel divergence are more than two times as frequent as expected by chance, meaning parallel divergence is also a significant feature of lineage II (*P* < 0.001).

## Discussion

Independent mutation accumulation in haplotypes of diploid asexual organisms is considered to be strong, direct support for evolution under obligate asexuality (19). Surprisingly, empirical evidence for this Meselson effect in parthenogenetic animals is, as yet, either lacking or equivocal [recently reviewed in Hoerandl et al. (25)]. Here, we report population genomic signatures supporting the presence of the Meselson effect in the long-term asexual oribatid mite species *O. nova*, namely: I) intraindividual variation exceeding between-population variation, II) a large proportion of conserved heterozygous variants shared among individuals of different lineages and geographic locations, III) separation of haplotypes rather than lineages in haplotype phylogenies, and IV) topologies of haplotype subtrees are matching. These signatures were absent in the sexual species *O. subpectinata*. Accordingly, transcriptome-wide heterozygosity was overall higher in *O. nova* than in *O. subpectinata* even though two individuals of *O. nova* featured very low heterozygosity. This study provides strong positive evidence for the Meselson effect in a parthenogenetic animal and thus long-term evolution in the absence of sex.

Hybridization at the origin of asexuality and divergence between paralogs can generate allele divergence patterns mimicking the Meselson effect even in recently evolved asexual species (6, 43). While we cannot formally exclude a hybrid origin of asexuality, it is unlikely to explain our results for three reasons: First, *O. nova* displays heterozygosity levels (1.05%) that are lower than those typically observed in parthenogenetic animals with a hybrid origin [ranging from 1.73% in the amazon molly *Poeciliopsis formosa* to 8.5% in the root knot nematode *Meloidogyne javanica* (44)] and much lower than the divergence from its sexual sister species, *O. subpectinata* (16.7%). Second, hybridization is unlikely to account for the high proportion of heterozygous SNPs private to the two lineages (exceeding the numbers of shared heterozygous SNPs among lineages by 293% and 11% in lineages I and II, respectively; Fig. 3) because this would require two independent, reciprocal hybridization events simultaneously generating two asexual lineages (*SI Appendix*, Fig. S4). Finally, a hybrid origin cannot account for the parallel divergence of haplotypes within lineages I and II; this can only be explained by the Meselson effect. Similarly, divergence between paralogs (18, 22, 23) is unlikely to explain our results since the phased regions showing the Meselson effect derived from genes present as single copies. Therefore, the observed haplotype divergence patterns are best explained by mutations that occurred after the origin of asexuality, which thus indicates long-term asexual evolution independently of possible hybridization at the origin of asexuality or paralog divergence. Dating the origin of asexuality in *O. nova* is difficult given that haplotype divergence is likely constrained via homogenizing mechanisms, and the observed haplotype divergence thus likely represents a fraction of the substitutions that occurred since the origin of asexuality. Moreover, estimating when sex was lost would require using mutation rates which have not yet been estimated for mites. Both factors prevent a useful verification of the previously suggested age of asexuality based on splits within *O. nova* [6–16 My (40)].

Our results indicate that levels of haplotype divergence vary strongly among genomic regions in *O. nova* individuals, as well as among different *O. nova* lineages (Fig. 4*A* and Table 1). This is in line with previous findings of varying levels of heterozygosity loss vs. retention in other asexual animal species (44) and could explain why previous studies using individual genes in asexual mites and ostracods found no increased heterozygosity (21, 45). Our results thus illustrate that haplotype divergence and other genomic consequences of asexuality need to be studied on whole genomes or transcriptomes rather than on a few genes [Neiman et al. (46)].

Besides haplotype divergence in *O. nova*, our study indicates the presence of coexisting, strongly divergent lineages with different heterozygosity levels (Fig. 2*A* and Table 1). Coexistence of strongly divergent lineages in *O. nova* has been shown previously based on the mitochondrial gene COI (separation of lineages was estimated to have occurred 6–16 Mya) and was considered to indicate coexistence of forest and grassland genotypes (40). *O. nova* occurs over a wide variety of habitat types and shows a cosmopolitan distribution (47), suggesting that the extensive intraspecific polymorphism might be linked to large population sizes (48) and dispersal capabilities (but note that studies on dispersal patterns in sexual compared with asexual oribatid mite species are lacking). Independently of the origin of the extensive polymorphism, we also observed differences in heterozygosity between lineages. These could be linked to different mutation rates between lineages or to the presence of nonmutually exclusive mechanisms of heterozygosity loss, including the meiotic parthenogenesis proposed for asexual oribatid mites, lineage-specific deletions [hemizygous genome regions (49)], and gene conversion mechanisms (50). One type of conversion mechanism, GC-biased gene conversion, has notably been suggested to contribute to the loss of heterozygosity in other parthenogenetic animals, e.g., in the darwinulid ostracod *Darwinula stevensoni* and in aphids of the tribe *Tramini* (51, 52). Finally, it is important to note that outside of the genome regions featuring the Meselson effect in *O. nova*, some form of cryptic or noncanonical sex cannot be excluded [e.g., Signorovitch et al. (8)]. Detecting the corresponding allele-sharing signatures across individuals [e.g., Vakhrusheva et al. (53)] would require different data and is a challenge for future studies.

Irrespective of the mechanisms underlying heterozygosity losses in *O. nova*, our results strongly support genome evolution in the absence of sex over evolutionary times in the asexual oribatid mite species *O. nova*. This is in line with previous studies that have shown that oribatid mites are able to overcome some major selective disadvantages predicted for asexual lineages. Unlike other asexual animal taxa, genomes of oribatid mites show reduced accumulation of slightly deleterious mutations compared to their sexual relatives, possibly facilitated by large population sizes (28, 44, 48, 54). Also, similar to other asexual organisms, oribatid mites show no increased abundance and activity of transposable elements compared to sexuals (55, 56). These findings suggest that asexual oribatid mites indeed escape the dead-end fate usually associated with asexual lineages.

## Methods

### Animal Sampling and DNA/RNA Extraction.

Animals were sampled in the fall of 2015 and 2017 from leaf litter and soil at four different forest sites in Germany (Göttingen forest [GF], Hainich [H], Kranichstein forest [KF], and Schwäbische Alb [SA]; for details, see *SI Appendix*, Table S6). Living animals were separated from the leaf litter with heat gradient extraction (57) and identified (58), followed by at least 1 wk of starving to reduce potential contaminants derived from gut contents. Afterward, animals were cleaned by removing surface particles in sterile water, several minutes of washing in a solution of hexane/bleach/detergent/water (25:25:1:49), and rinsing with sterile water before extraction. Note that animals were alive after cleaning.

For generating reference genomes, DNA was extracted from one single individual of *O. subpectinata* collected in 2015 from GF leaf litter and *O. nova* collected in 2015 from KF leaf litter using the QIAamp DNA Micro kit according to the manufacturer’s instructions. To generate transcriptomes for annotation of reference genomes, RNA was extracted from five pooled individuals per species from the same collection batch. For this, individuals were frozen in liquid nitrogen and, after addition of TRIzol (Life Technologies), mechanically crushed with beads (Sigmund Lindner). Next, chloroform and ethanol were added to the homogenized tissue and the aqueous layer transferred to RNeasy MinElute Columns (Qiagen). Subsequent steps of RNA extraction were done following the RNeasy Mini Kit protocol, including DNase digestion. Finally, RNA was eluted into water and stored at −80 °C. To infer haplotype divergence, RNA was extracted from single individuals of *O. nova* and *O. subpectinata* from H, SA, and KF (three individuals from each forest site for each species) from the 2017 collection batch. RNA extraction was done as described above. DNA and RNA quantity and quality were measured using, respectively, Qubit and NanoDrop (Thermo Scientific), and Bioanalyzer (Agilent).

### Reference Genome Assemblies and Contaminant Removal.

For genome sequencing, extracted DNA from single individuals was amplified in two independent reactions using the SYNGIS TruePrime WGA kit and then pooled. Four libraries were generated for each reference genome (three paired end libraries with average insert sizes of 180, 350, and 550 bp, respectively, and a mate-pair library with 3,000-bp insert size). Libraries were prepared using the Illumina TruSeq DNA or Nextera Mate Pair Library Prep Kits, following manufacturer instructions, and sequenced on the Illumina HiSeq 2500 system, using v4 chemistry and 2× 125-bp reads at FASTERIS SA. This resulted in a total number of 451*10^6^ reads for *O. nova* with a total read coverage of 490-fold and 387*10^6^ reads for *O. subpectinata* with a total read coverage of 420-fold (for details, see *SI Appendix*, Table S2). Read quality trimming and adapter clipping of paired reads were done using Trimmomatic v0.36 (59) with the following options: ILLUMINACLIP:/all-PE.fa:2:30:10 LEADING:20 TRAILING:20 SLIDINGWINDOW:3:20 MINLEN:100. This resulted in 56% and 46% surviving read pairs (for details, see *SI Appendix*, Table S2). For mate pair quality trimming, Nxtrim v0.4.1 (60) with options–separate–preserve-mp–minlength 40, followed by Trimmomatic v0.36 with options ILLUMINACLIP:/all-PE.fa:2:30:10 LEADING:20 TRAILING:20 SLIDINGWINDOW:4:20 MINLEN:60 were used to identify properly paired reads and to remove low-quality bases and adapters. This resulted in 54% and 48% surviving read mate pairs (for details, see *SI Appendix*, Table S2).

With the available read data, we tested a range of assembly strategies. The best assemblies were generated using normalized overlapped reads, because whole-genome amplification introduces overrepresented genomic regions, which leads to coverage bias that is problematic for assembly. Overlapped read libraries were generated by merging the paired forward and reverse reads of the 180-bp read libraries and additionally merging unpaired reads, followed by normalization using BBnorm v37.82 (61). These normalized overlap read libraries were assembled into contigs using SPAdes v3.10.1, a multi k-mer assembler (62), with options -m 400–careful -k 21, 33, 55, 77, 99, 111, 127. The resulting contigs were ordered into scaffolds using the 350-, 500-, and 3,000-bp read libraries using SSPACE v3.0 (63) with default parameters. To close gaps emerging during scaffolding, GapCloser v1.12 (64) with option -l 125 was run. For details, see https://github.com/AsexGenomeEvol/HD_Oppiella: assembly and mites.

Scaffolds that were likely from contaminants (e.g., bacteria, fungi) were removed by first annotating and visualizing contaminations using BlobTools v1.0 (65), followed by custom filtering. For this, coverage of each scaffold was estimated by mapping reads back to the scaffolds using bwa mem v0.7.15 (66) and coverage calculated with BBTools v73.82 (61). Additionally, for annotation, scaffolds were blasted using ncbi-blast v2.7.1+ blastn with options -outfmt '6 qseqid staxids bitscore evalue std sscinames sskingdoms stitle' -max_target_seqs 10 -max_hsps 1 -evalue 1e-25 against the nt database v 2016–06. Scaffolds without hits to metazoans were filtered out from the assemblies using a custom script (see https://github.com/AsexGenomeEvol/HD_Oppiella: contamination_filtration.py). Next, scaffolds were sorted by decreasing length, scaffold headers renamed and scaffolds shorter than 500 bp removed, resulting in the final assemblies (v03). The assemblies were checked for quality and completeness by calculating standard genome statistics and by checking presence, fragmentation, and duplication of arthropod core genes using CEGMA v2.5 and BUSCO v3.0.2 (67, 68). For details, see *SI Appendix*, Table S1.

### Genome Annotation.

The de-contaminated genome assemblies v03 were annotated using MAKER v2.31.8 (69), a hybrid de novo evolution-based and transcript-based method. For this, repetitive genomic regions are first masked using RepeatMasker v4.0.7 (70) as implemented in MAKER. Protein coding genes were then predicted in a 2-iterative way described in Campbell et al. (71) with minor modifications following author recommendations. For the first iteration, genes were predicted using Augustus v3.2.3 (72) trained with the BUSCO v3.0.2 results (arthropoda_odb9 lineage with the–long option). A combination of UniProtKB/Swiss-Prot (release 2018_01) and the BUSCO arthropoda_odb9 proteome were used as protein evidence. The Trinity assembled mRNA-seq sequences (described below) were used as transcript evidence. The resulting gene models from iteration 1 were then used to retrain Augustus as well as SNAP v2013.11.29 (73) and a second iteration was performed. Subsequently, predicted protein coding genes were functionally annotated using Blast2GO v5.5.1 (74) with default parameters against the NCBI *nonredundant arthropods* protein database and the *Drosophila melanogaster* (drosoph) database v 2018–10. The MAKER configuration files are available at https://github.com/AsexGenomeEvol/HD_Oppiella.

For the MAKER annotation, RNA-seq reads were quality trimmed with Trimmomatic v0.36 with options adapters.fa:2:30:12:1:true LEADING:3 TRAILING:3 MAXINFO:40:0.4 MINLEN:80. For generating genome-guided transcriptome assemblies, trimmed reads were first mapped against the genomes using STAR v2.5.3a (75) under the “2-pass mapping” mode and default parameters. Following, the outputs were used with Trinity v2.5.1 (76) set to “genome guided” mode (parameters:–genome_guided_max_intron 100000–SS_lib_type RF–jaccard_clip). For quality filtering of the resulting transcriptomes, the trimmed RNA-seq reads were mapped back against the transcriptomes using Kallisto v0.43.1 (77) with options–bias and–rf-stranded, then transcripts with at least 1 TPM in any samples were retained. All computation for genome assembly and annotation were run on the Vital-IT cluster of the Swiss Institute of Bioinformatics.

### Pairwise Divergence between Sister Species.

Transcript sequences were reconstructed from annotated reference genomes using GffRead (option -w) (78). Single-copy orthologs were identified using Orthofinder [standard parameters; option -d (79)], aligned using muscle [standard parameters (80)], positions including any gaps removed [trimal -nogaps (81)], and pairwise divergence calculated using Geneious [p-distance (82)].

### Haplotype Divergence: RNA-seq, Quality Control, and Mapping.

RNA extracts were fragmented to 175 nt for strand-specific library preparation using the NEBNext Ultra II Directional RNA Library Prep Kit. Paired-end sequencing with a read length of 100 bp was performed on a HiSeq 2000 platform at the Genomics Technology Facility (Lausanne, Switzerland). Data processing for haplotype divergence inference was done using the high-performance computing cluster of the Gesellschaft für Wissenschaftliche Datenverarbeitung (Göttingen, Germany). RNA-seq reads were adapter- and quality-trimmed using TrimGalore v0.6.5 with default options (Phred quality threshold 20; adapter auto-detection) (83, 84). Contaminating sequences were removed using kraken2 (–paired;–db minikraken2_v2) (85) followed by mapping paired-end reads of each individual simultaneously against their respective reference genome, scaffolds flagged as contaminating sequences assembled together with the respective mite reference genomes (identified as described above), the human reference genome GRCh38.p12 (GenBank assembly accession: GCA_000001405.27), and the human microbiome (downloaded from https://www.hmpdacc.org/hmp/HMREFG/all/index.php) using bbmap v37.66 (bbsplit; maxindel = 100k; ambiguous = best) (61). Portions of reads were found to be derived from contaminating RNA of human and microbial origin with fractions ranging from 40.36 to 90.31% in *O. subpectinata* and 53.33 to 93.04% in *O. nova* (*SI Appendix*, Table S7). Only oribatid-mite-exclusive reads, i.e., read pairs that mapped best against the mite reference genomes, were kept for further processing and mapped to the reference genomes using STAR v2.7.3a with standard parameters. All commands are available under https://github.com/AsexGenomeEvol/HD_Oppiella: mapping.U.

### Haplotype Divergence: Variant Calling.

Read-group information was added and PCR and optical duplicates were removed from mapped reads using Picard v2.20.2 (86). Reads without a mapping mate were deleted using samtools view (87) and reads sorted by coordinate using GATK v4.0.3.0 SortSam (88). Next, the nine thus-filtered alignments per species were merged with samtools merge for subsequent SNP calling. Sequences spanning intronic regions were removed using GATK SplitNCigarReads. GATK HaplotypeCaller was run per individual with -ERC set to BP_RESOLUTION to enable calling of nonvariant sites and –dont-use-soft-clipped-bases to exclude soft-clipped overhangs from SplitNCigarReads. Individual gvcf-files were combined into one species-gvcf-file using GATK CombineGVCFs. Genotypes were called using GATK GenotypeGVCFs and the option -allSites. All commands used are available under https://github.com/AsexGenomeEvol/HD_Oppiella: calling.U

### Multidimensional Scaling Plots and AMOVA.

For calculating multidimensional scaling (MDS) plots and AMOVA, first genotypes with a coverage <10 were removed from gvcf-files using vcftools v0.1.15 (89). Next, sites including at least one missing genotype, monomorphic or triallelic variants, and indels were removed. To visualize genotype composition of populations, MDS (two scales for two-dimensional representation) was done using plink v1.9 with the options–cluster,–mds-plot 2 eigvals and –allow-extrachr (90). Population differentiation was tested based on the filtered set of SNPs with an analysis of molecular variance (AMOVA) and a randomness test with the packages vcfR and poppr in R (91–94). All commands used are available under https://github.com/AsexGenomeEvol/HD_Oppiella: MDS.U, AMOVA.R.

### Heterozygosity, F_is_, and SFS.

For calculating the percentage of heterozygous genotypes per individual, variants were filtered as described above (except monomorphic variants were not excluded). The percentage of heterozygous positions per individual was calculated using unix commands. Similarly, for F_is_ and SFS calculation, first genotypes with a coverage <10 were removed from gvcf files using vcftools v0.1.15. For F_is_ calculation, the gvcf-file was next subset into lineages I and II for *O. nova* and populations for *O. subpectinata* using vcf-subset (vcftools). For SFS calculation, subsetting was done for seven individuals of *O. nova* potentially showing the Meselson effect (*Results*) with F_is_ < 0. Afterward, sites including at least one missing genotype, monomorphic or triallelic variants, and indels were removed from the different subsets. F_is_ was calculated based on the filtered sets of SNPs using vcftools (option–het). The SFS was calculated using Pop-Con and standard parameters (95). The fold excesses of shared heterozygous SNPs over HWE were estimated by running Pop-Con with the option -fold for a range of parameters and comparing the specific genotype profiles for an indication of excess (shown as part of expected SFS). All commands used are available under https://github.com/AsexGenomeEvol/HD_Oppiella: heterozygosity.U, Fis.U and SFS.U.

### Haplotype Phasing.

For phasing, variants were filtered as described above (except only sites completely missing any genotype information, i.e., all individuals missing a genotype, were excluded). Phasing of haplotypes was done per individual using phASER v1.1.1 with minimum mapping quality of reads set to 30, minimum base quality set to 20, and bottom cutoff to quantile for alignment score set to 0 in paired-end mode for each individual, separately, utilizing heterozygous variants with minimum coverage of 10 for each individual (96). Haplotypes with <10 unique reads mapping were removed from the output data. Output data from phASER were converted into haplotype sequences for the corresponding positions in the reference genome using a custom script (available under https://github.com/AsexGenomeEvol/HD_Oppiella: meselson.py), which extracts the corresponding sequence from the reference genome and generates the two haplotypes by modifying the extracted sequence with the haplotype information from phASER. Furthermore, as some SNPs might not have been called by GATK HaplotypeCaller due to insufficient coverage, all bases of the reference genome with coverage <10 (the coverage threshold for genotype filtration) were excluded from further analysis (changed to N; coverage estimated with bedtools version 2.26.0 from individual bam-files after removing overhanging N’s at read ends using GATK SplitNCigarReads with the option–process-secondary-alignments). All regions comprising at least one phase per individual that overlapped with a phase of a different individual by at least 100 bp were included for downstream analyses (forming continuous stretches of phases; see *Results*). Haplotypes were labeled according to their divergence from the reference genome (haplotype A being closer to the reference genome, haplotype B being more diverged). For this, positions including degenerate bases were deleted using trimAl (81) and the pairwise distance of each haplotype to the reference genome was calculated using snp-dist (97). Only thus modified phaseable regions ≥100 bp were included for downstream analyses. All commands and two scripts used are available under https://github.com/AsexGenomeEvol/HD_Oppiella: phasing.U, refgenomedist.U, extract.pl and convert_fasta.py.

To identify putative paralogs in the phased regions, these regions were tested for double coverage compared to the genomic baseline. Reads used to assemble genomes were mapped back to single-copy genes and duplicated genes identified by BUSCO (see above), and additionally to the phased regions and to scaffolds from which the phased regions were derived (but that were masked in the phased regions), using bowtie2 v2.3.4.1 with standard parameters (98). The mapped alignments were quality filtered (MAPQ score > 10) using samtools and optical and PCR duplicates removed using Picard Tools Mark Duplicates v2.22.0. Following, coverage was calculated using bedtools genomecov v2.26.0 (99).

### Topology Testing.

To enable testing whether alignments of phaseable regions are better explained by a topology separating the haplotypes (as expected under asexuality) compared to a topology separating populations (expected under sexual reproduction) or vice versa, a constrained tree search was done. Two constrained ML trees, one complying with a fixed haplotype-separating topology (asex-tree), the other with a fixed population-separating topology (sex-tree), were calculated for each phased region using iqtree v1.6.10 with 1,000 bootstrap replicates and model-testing included (100). For *O. nova,* we restricted the analysis to the seven individuals representing the two divergent lineages (*Results* and Fig. 2*A*). The *O. nova* asex-trees were constrained to separate the haplotypes A and B at its base, lineages I and II per haplotype, and finally the populations per lineage and haplotype (for the constraining tree, see https://github.com/AsexGenomeEvol/HD_Oppiella: Onasex.tre). The sex-trees were constrained to separate the lineages I and II at its base and the populations per lineage (no haplotype separation; for the constraining tree, see https://github.com/AsexGenomeEvol/HD_Oppiella: Onsex.tre). For *O. subpectinata*, the asex-trees were constrained to separate the haplotypes A and B at its base and the populations per haplotype (to provide an unrooted tree including a trichotomy the haplotypes B of the most divergent population SA were separated from haplotypes B of the other two populations; for the constraining tree, see https://github.com/AsexGenomeEvol/HD_Oppiella: Osasex.tre). The sex-trees were constrained to separate exclusively the populations (no haplotype separation; for the constraining tree, see https://github.com/AsexGenomeEvol/HD_Oppiella: Ossex.tre). Next, the distance between the two resulting trees and an unrestricted best-fitting ML tree was estimated according to Kuhner and Felsenstein (42) with the dist.topo function implemented in the R package ape (101). To enable the comparison between distances of different phaseable regions, the topological distances of the best-fitting ML tree to the haplotype-separating tree and to the population-separating tree were combined as Δ _dist._ _asex-tree__-__dist._ _sex-tree_ value for each phaseable region. To test whether the haplotype-separating tree was a significantly better fit to the alignment than the population-separating tree, trees were compared using RELL approximation with 10,000 RELL replicates and an approximately unbiased (AU) test with iqtree (102, 103). Using the AU test, we also compared both constrained trees to the unconstrained tree. For detailed information, see https://github.com/AsexGenomeEvol/HD_Oppiella: treecalcandtopotest.U and topodist.UR.

### Parallel Divergence Testing.

Phasing and haplotype reconstruction were done as described above but coverage was reduced to a minimum of five to increase the number of informative sites, and thereby the number of nonpolytomous trees. Calculation of best-fitting ML trees was done as described above. Resulting topologies were screened by eye for being nonpolytomous, for showing haplotype separation and for parallel divergence. To calculate the probability of observing parallel divergence in at least eight trees out of 31 haplotype separating eight taxa trees by chance (lineage I), we used the binomial formula: $\;\sum_{k}^{n}\left(n!/\left(\left(n-k\right)!k!\right)\right)x^{k}\cdot y^{n-k}$ with *n* = 31 (31 trees showing haplotype separation), *k* = 8 (8 trees showing parallel divergence), *x* = 0.067 (the probability to observe parallel divergence in two rooted four taxa trees by chance), and *y* = 0.933 (counter event; 1 − *x*). To calculate the probability to observe parallel divergence in at least 38 trees out of 55 haplotype separating six taxa trees by chance (lineage II), we used the binomial theorem with *n* = 55, *k* = 38, *x* = 0.333, and *y* = 0.667.

### Statistical Analyses.

All statistical analyses were done in R, version 3.6.3 (91), unless mentioned otherwise.

## Supplementary Material

Supplementary File

## Data Availability

Reference genomes and annotations of the two species have been deposited in the European Nucleotide Archive (ENA) (accession number PRJEB39968). Transcriptomes and RNA-seq reads of the two species used for inferring allelic sequence divergence and for annotations have been deposited in the Sequence Read Archive (SRA) (accession number PRJNA662767). All scripts and commands as referred to in the article have been deposited in the GitHub repository (https://github.com/AsexGenomeEvol/HD_Oppiella).
