# Supplementary material for: Haplotype divergence supports long-term asexuality in the oribatid mite Oppiella nova
Source: Proc Natl Acad Sci U S A. 2021 Sep 17;118(38):e2101485118. doi: 10.1073/pnas.2101485118 (PMC8463897; doi:10.1073/pnas.2101485118)
Supplement: Supplementary File [file pnas.2101485118.sapp.pdf]

Supplementary Information

Supplementary Figures

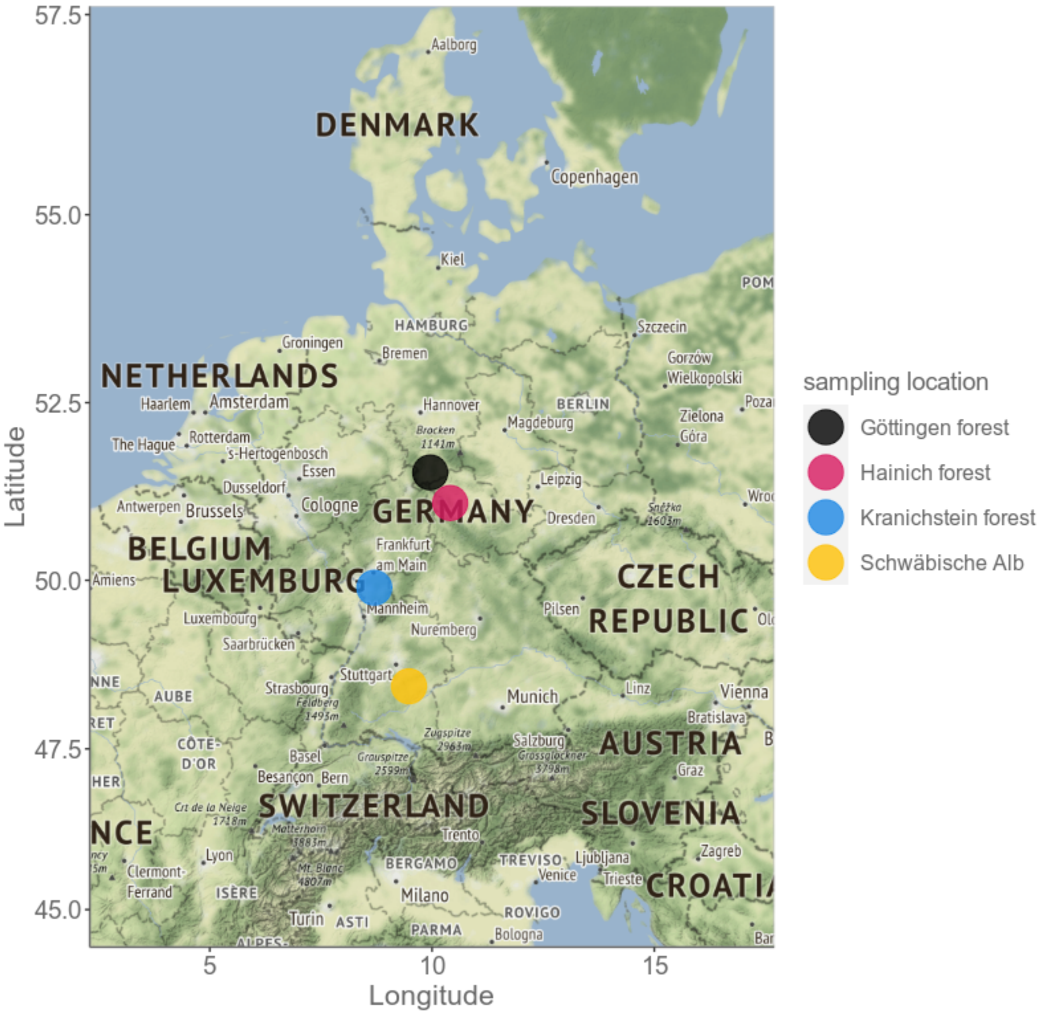

Supplementary Figure 1: The oribatid mite samples were collected in different forests in Germany. For detailed information on sampling, see Supplementary Table S6.

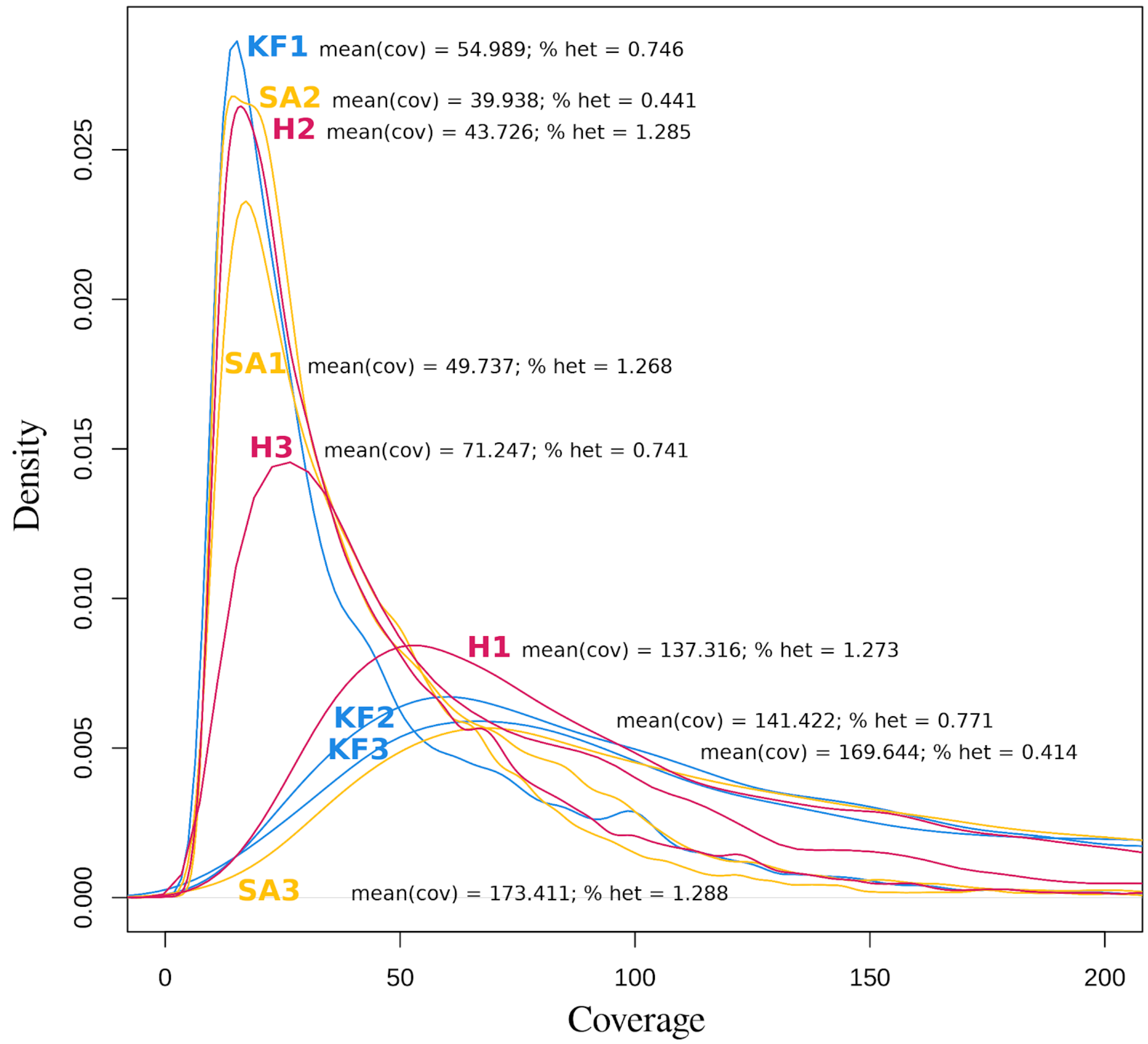

Supplementary Figure 2: Kernel density of genotype coverage per individual. There is no obvious correlation between percentages of heterozygous sites (% het) and mean coverage of genotypes (mean(cov)) per individual indicating that the large differences in heterozygosity between individuals are not driven by coverage variation. H Hainich; KF Kranichstein forest; SA Schwäbische Alb.

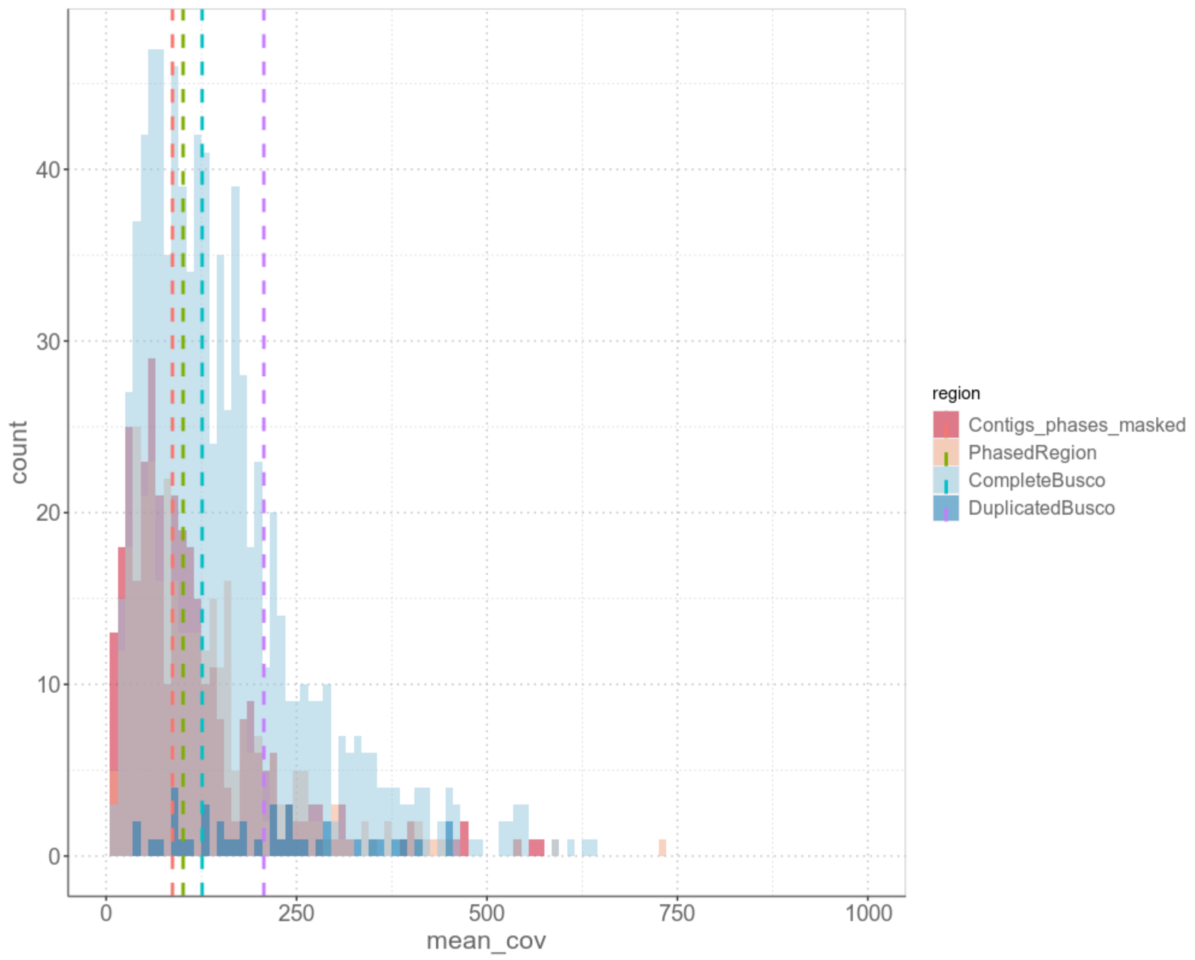

Supplementary Figure 3: Coverage distributions indicate that phased regions are not enriched for merged paralogs. Count frequencies of coverages of the different regions based on genomic read data: contigs that contained the phased regions but were masked for these, the phased regions, complete single-copy genes and duplicated genes as identified by BUSCO. Dotted lines represent the median of the coverage for the respective regions.

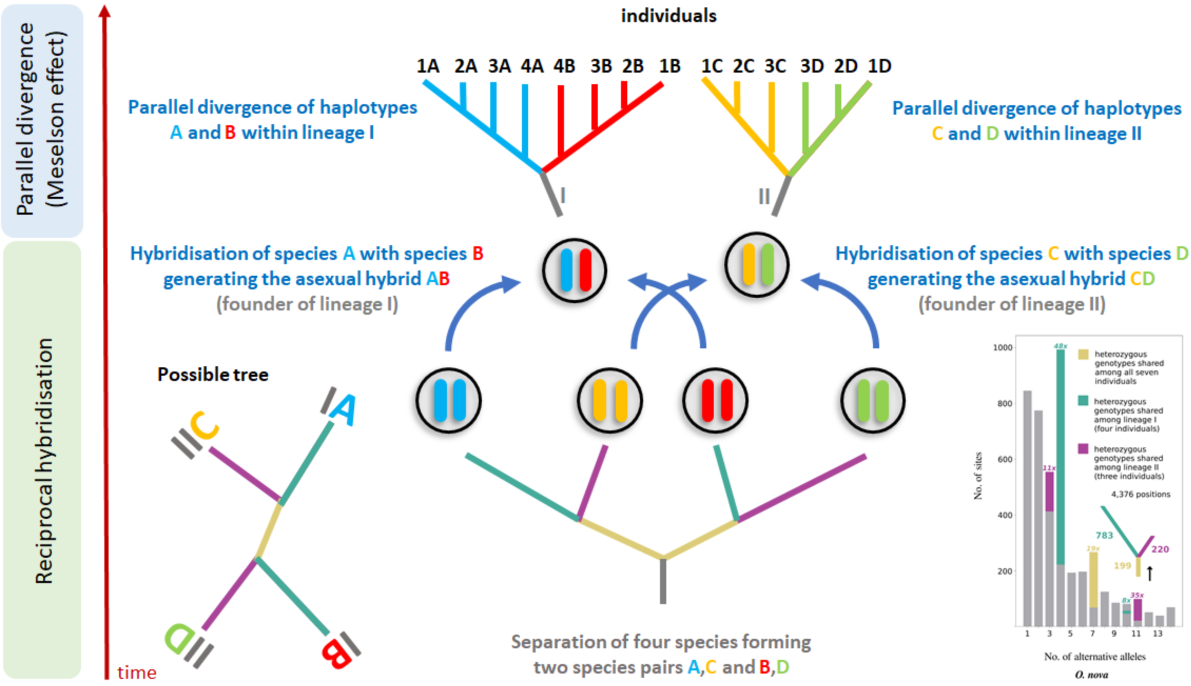

Supplementary Figure 4: Hypothetical scenario of the "Meselson effect on top of reciprocal hybridisation".

Reciprocal hybridisation: separation of four species forming two species pairs A,C and B,D is followed by two independent "reciprocal" hybridisation events. The two hybridisation events generate two asexual hybrids (AB and CD) which serve as founders of lineages I and II. Under this scenario, the initial separation between the species pairs (yellow metallic branches) generates shared heterozygous SNPs (yellow metallic bar in the site frequency spectrum; see Figure 3) after hybridisation while separation within species pairs (purple and turquoise branches) generates lineage-specific (private) heterozygous SNPs (purple and turquoise bars) after hybridisation. Consequently, a tree would separate haplotypes at its basis (in this case haplotype pairs A,C vs B,D) followed by separation of lineages I and II.

Parallel divergence (Meselson effect): after hybridisation and the loss of sex, the haplotypes within lineages start to diverge in parallel, i.e. haplotype A diverges in parallel with haplotype B and C with D (Meselson effect). While we cannot exclude two independent reciprocal hybridisation events that generated the shared and lineage specific heterozygous variants, our finding of parallel divergence within lineages can only be explained by the Meselson effect.

Supplementary Tables

Supplementary Table S1: Genome statistics and completeness scores. v02: decontaminated assembly, v03: decontaminated assembly with > 500bp scaffold length size filter

Species	CEGMA	Busco	Genome Size [Mbp]	Scaffold N50 [bp]	Number of scaffolds	Largest scaffold length [bp]	GC content [%]	N [%]
O. nova			207.08	6604	83865			1.64
Blobtools v02	C: 88.31 P: 95.56	C:87.1%[S:78.8%, D:8.3%],F:6.6%, M:6.3%,n:1066	202.44	6493	82784	406770	30.41	1.68
Filtered v03	C: 88.31 P: 95.56	C:87.5%[S:78.9%, D:8.6%],F:6.6%, M:5.9%,n:1066	196.72	6753	63118	406770	30.37	1.73
O. subpectinata			217.82	6873	73173			1.44
Blobtools v02	C: 88.31 P: 97.58	C:86.6%[S:78.6%, D:8.0%],F:6.3%, M:7.1%,n:1066	217.02	6852	73101	165769	30.84	1.44
Filtered v03	C: 88.31 P: 97.58	C:86.2%[S:78.3%, D:7.9%],F:6.4%, M:7.4%,n:1066	213.17	7017	60250	165769	30.83	1.47

Supplementary Table S2: Information on the read library insert sizes, number of reads generated, estimated coverage, and number of surviving read pairs after filtering.

Species	Library	No of raw read pairs	Coverage	No of read pairs surviving
O. nova	180	125889255	137	95460017 (75.83%)
	350	73798941	80	44494871 (60.29%)
	550	76545499	83	34976565 (45.69%)
	3000	174946393	190	33642006 (53.99%)
O. subpectinata	180	112721137	122	89023834 (78.98%)
	350	87673896	95	65281720 (74.46%)
	550	71743398	78	40465571 (56.40%)
	3000	115207485	125	21898456 (53.96%)

Supplementary Table S3: Annotation statistics

Species	Gene number	mRNA number	Gene min length [bp]	Gene mean length [bp]	Gene max length [bp]	Genome covered by genes [%]	Exon mean length [bp]	Intron mean length [bp]
O. nova	23761	24130	30	1956	42876	23.6	258	270
O. subpectinata	23555	23803	49	2148	53142	23.7	258	246

Supplementary Table S4: Detailed information on successfully phased regions. * No. phased regions after removal of sites with coverage < 10; # No. phased regions after removal of regions with sequence similarity too large for tree calculation; ~ No. significantly haplotype divergence positive regions.

Species	No. phased regions	total length	*	total length	median length	#	total length	~	total length
O. nova	329	1,233,469	281	140,966	358	223	127,123	69	37,693
O. subpectinata	302	2,010,902	275	206,255	563	268	204,851	1	115

Supplementary Table S5: Results of ML based tree topology (AU) tests. The table lists numbers of haplotype sequence alignments of phased regions that provide a significantly better fit to a constrained tree separating haplotypes than to a constrained tree separating populations (asex-tree > sex-tree) and vice versa (sex-tree > asex-tree) for the asexual species *O. nova* and its sexual relative *O. subpectinata*. To further assess how consistent the topology of the best ML tree was with either one of the constrained trees, we additionally ran AU tests using the unconstrained tree (e.g. unconst. = asex-tree > sex-tree indicates non-significant differences between the unconstrained tree and the asex-tree but rejection of the sex-tree). For some phased regions AU tests could not be run due to insufficient variation between haplotypes.

	O. nova	***O. subpectinata***
**asex-tree > sex-tree**	69	1
**sex-tree > asex-tree**	70	258
**non-significant**	70	4
**test impossible**	14	5
**unconst. = asex-tree > sex-tree**	12	0
**unconst. = sex-tree > asex-tree**	10	95

Supplementary Table S6: Additional information on sampling sites and species.

Sampling site	GPS coordinates	Species for WGS and transcriptome annotation (sampled fall 2015)	Species for RNAseq (sampled fall 2017)
**Goettingen forest**	51.533778,9.959861	*O. subpectinata*	
**Hainich**	51.104556,10.408500		*O. subpectinata*
			O. nova
**Kranichstein forest**	49.892194,8.701889	*O. nova*	*O. subpectinata*
			O. nova
**Schwäbische Alb**	48.442389,9.482250		*O. subpectinata*
			O. nova

Supplementary Table S7: numbers of read pairs/reads after different steps of quality trimming, contamination removal and mapping**.**

Species	Population	Indi-vidual	No. raw read pairs	No. read pairs after trimming	No. read pairs after contaminant removal	% contaminating read pairs	No. mapped reads after duplicate removal
O. nova	Hainich	H1	12,305,768	10,922,239	5,073,080	53.33	1,538,502
		H2	11,653,023	10,150,566	1,546,946	84.76	434,144
		H3	17,293,884	14,456,993	3,959,499	72.61	866,110
	Kranichstein forest	KF1	14,818,283	12,790,718	1,426,174	88.85	444,750
		KF2	14,514,107	12,380,574	4,874,135	60.63	1,780,426
		KF3	22,424,703	20,094,950	7,214,778	64.1	2,138,804
	Schwäbische Alb	SA1	10,460,975	9,570,865	1,339,805	86	567,076
		SA2	21,489,919	19,714,647	1,371,202	93.04	500,764
		SA3	23,061,245	21,030,385	7,930,318	62.29	2,083,292
O. subpectinata	Hainich	H1	21,756,064	20,776,464	5,399,072	74.01	2,828,074
		H2	18,105,719	15,780,873	5,727,395	63.71	1,320,928
		H3	12,584,357	11,011,539	2,464,755	77.62	997,412
	Kranichstein forest	KF1	26,438,129	23,518,688	4,829,639	79.46	3,472,828
		KF2	36,588,456	34,945,277	3,386,946	90.31	4,238,092
		KF3	28,975,922	23,341,626	11,877,834	49.11	3,279,550
	Schwäbische Alb	SA1	21,616,073	20,271,910	12,090,364	40.36	4,230,422
		SA2	12,186,958	10,229,333	2,695,017	73.65	739,384
		SA3	15,924,247	13,972,229	3,810,147	72.73	1,178,238
